# Melanocortins Contribute to Sequential Differentiation and Enucleation of Human Erythroblasts via Melanocortin Receptors 1, 2 and 5

**DOI:** 10.1371/journal.pone.0123232

**Published:** 2015-04-10

**Authors:** Eriko Simamura, Tomohiro Arikawa, Takayuki Ikeda, Hiroki Shimada, Hiroki Shoji, Hiroko Masuta, Yuriko Nakajima, Hiroki Otani, Hideto Yonekura, Toshihisa Hatta

**Affiliations:** 1 Department of Anatomy, Kanazawa Medical University School of Medicine, Uchinada, Ishikawa 920–0293, Japan; 2 Department of Biology, Kanazawa Medical University School of Medicine, Uchinada, Ishikawa 920–0293, Japan; 3 Department of Biochemistry, Kanazawa Medical University School of Medicine, Uchinada, Ishikawa 920–0293, Japan; 4 Department of Developmental Biology, Faculty of Medicine, Shimane University, Izumo 693–8601, Japan; Boston University School of Medicine, UNITED STATES

## Abstract

In this study, we showed that adrenocorticotropic hormone (ACTH) promoted erythroblast differentiation and increased the enucleation ratio of erythroblasts. Because ACTH was contained in hematopoietic medium as contamination, the ratio decreased by the addition of anti-ACTH antibody (Ab). Addition of neutralizing Abs (nAbs) for melanocortin receptors (MCRs) caused erythroblast accumulation at specific stages, i.e., the addition of anti-MC2R nAb led to erythroblast accumulation at the basophilic stage (baso-E), the addition of anti-MC1R nAb caused accumulation at the polychromatic stage (poly-E), and the addition of anti-MC5R nAb caused accumulation at the orthochromatic stage (ortho-E). During erythroblast differentiation, ERK, STAT5, and AKT were consecutively phosphorylated by erythropoietin (EPO). ERK, STAT5, and AKT phosphorylation was inhibited by blocking MC2R, MC1R, and MC5R, respectively. Finally, the phosphorylation of myosin light chain 2, which is essential for the formation of contractile actomyosin rings, was inhibited by anti-MC5R nAb. Taken together, our study suggests that MC2R and MC1R signals are consecutively required for the regulation of EPO signal transduction in erythroblast differentiation, and that MC5R signal transduction is required to induce enucleation. Thus, melanocortin induces proliferation and differentiation at baso-E, and polarization and formation of an actomyosin contractile ring at ortho-E are required for enucleation.

## Introduction

The differentiation of *ex vivo*-cultured red blood cells (RBCs) from human CD34^+^ hematopoietic stem cells was achieved by co-culturing with stromal cells [1]. Miharada *et al*. reported that terminal erythroid cells could be differentiated from CD34^+^ cord blood cells without feeder cells [2]. Erythropoiesis is regulated by soluble factors such as erythropoietin (EPO), stem cell factor (SCF) and interleukin (IL)-3 [1,3,4]. In addition, extracts of plasma components are required for culturing human erythroid cells, suggesting that unknown essential humoral factors are necessary for erythroid differentiation [2].

Signals transduced by the EPO receptor in erythroblasts induce phosphorylation in the MAPK/ERK [5], JAK2/STAT5 [6–8], and PI3K/AKT [9,10] pathways. It has been shown that an ERK-derived signal plays a dominant role in EPO-induced proliferation [11,12]. STAT5 knockout mice exhibit severe anemia because the expression of the antiapoptotic genes *Mcl-1* and *Bcl-xL* is deregulated, and the expression levels of iron regulatory protein 2 (IRP-2) and transferrin receptor 1 (CD71) are reduced [13]. PI3K/AKT activity is required for the regulation of cell polarization for enucleation [14].

Erythroid enucleation is the critical step for terminal differentiation in erythropoiesis. Enucleation has been thought of as an event of asymmetric cell division [15,16]. When examining the intracellular mechanisms for enucleation, reports have determined that the Rac GTPases and mDia2, a RhoA and Rac effector, pathway drives the formation of contractile actomyosin rings [17]. Phosphorylated myosin light chain 2 (MLC2) is assembled into a contractile actomyosin ring in a population of enucleating erythroblasts [18]. Moreover, non-muscle myosin IIB is required in the enucleation of human erythroblasts [19]. However, although several findings regarding the intracellular mechanisms of enucleation have been reported, the extracellular enucleation factors remain unknown.

Adrenocorticotropic hormone (ACTH) is derived from the post-translational processing of the precursor protein proopiomelanocortin in the anterior lobe of the pituitary gland and the placenta. Alpha-melanocyte-stimulating hormone (α-MSH; ACTH1–13) is processed in the hypothalamus, intermediate lobe of the pituitary gland, skin, and placenta [20,21]. In contrast to the levels in the pituitary gland, α-MSH levels are almost equal to ACTH levels in the placenta [22]. Melanocortin receptors (MCRs) consist of five members, and the affinity of MCRs with ACTH, α-MSH, β-MSH, andγ-MSH have been confirmed [23–27]. In adults, MCRs have been reported to be expressed in lymphocytes, macrophages [28], and neutrophils [29].

In previous studies, we showed that MC2R and MC5R are expressed in fetal nucleated RBCs in mice and rats [30,31]. However, the role of MC2R and MC5R in fetal nucleated RBCs remains unknown. When we investigated whether placental ACTH induces leukemia inhibitory factor secretion from fetal nucleated RBCs in rats [31], we unexpectedly found a large number of nuclei exhausted from nucleated erythrocytes in the culture media after supplementation of ACTH. From this observation, we speculated that placental ACTH participated in the enucleation of erythroblasts. The level of ACTH, secreted from the placenta increases in rat fetal serum at 14.5 days post coitum [31], when erythroblasts mature into enucleated RBCs [32]. The terminal maturation and enucleation of primitive RBCs occurs between 7 weeks and 10 weeks in the first trimester placenta [33]. In the present study, we revealed a regulatory mechanism of the melanocortin–MCR system in human erythropoiesis and proposed the idea that melanocortins are novel and essential factors for erythroblast differentiation.

## Materials and Methods

### Cell culture

**Fig 1:**
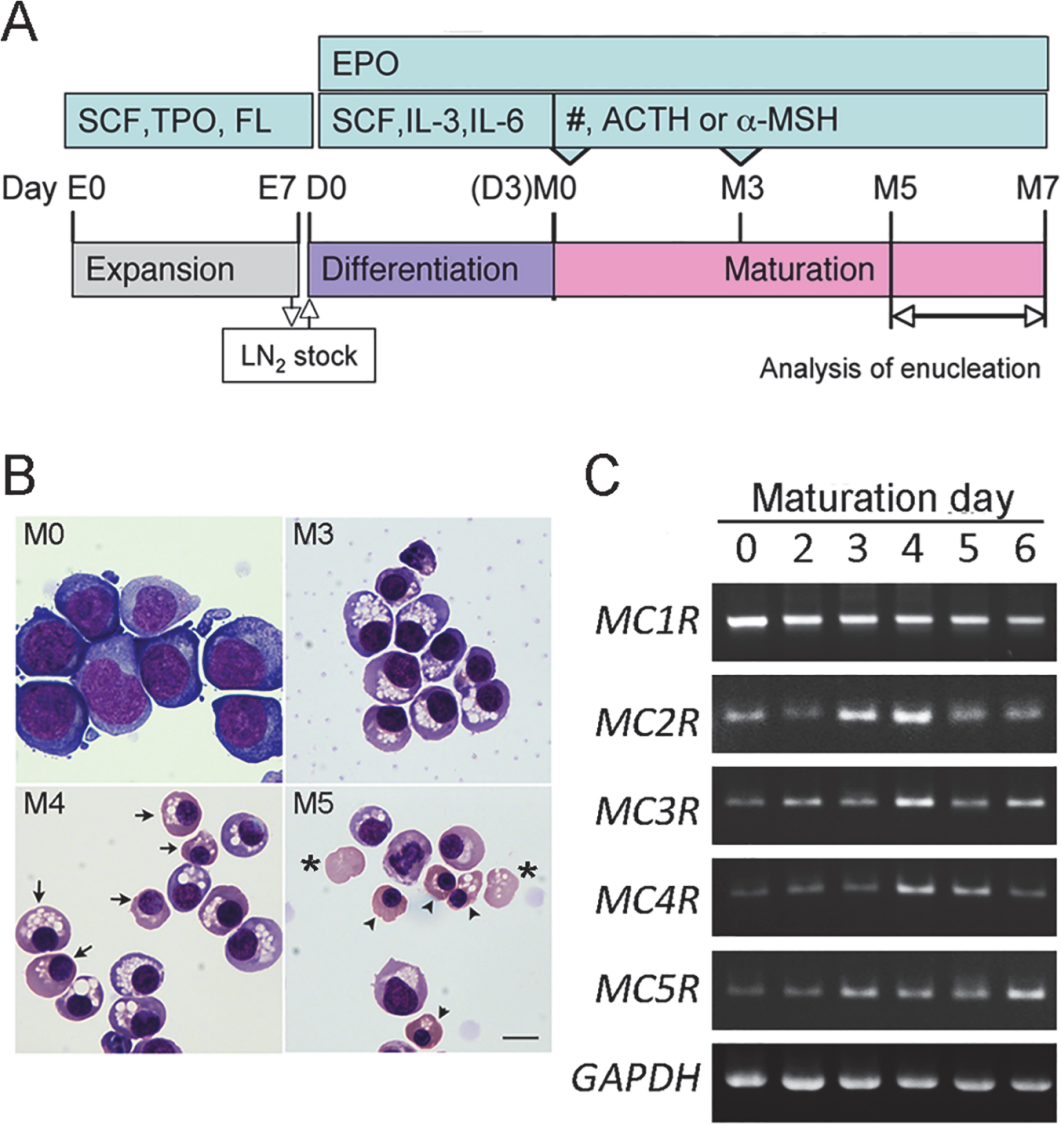
MCR expressions during erythroblast differentiation. (**A**) Schematic diagram of an *in vitro* differentiation protocol for deriving erythroblasts from human HPCs. Human CD34^+^ HPCs were expanded for 7 days (E0–E7) and stocks were frozen in liquid nitrogen (LN_2_). The stock cells were differentiated for 3 days (D0–D3) before undergoing maturation (M0–M7). The number of enucleated erythrocytes increased from M5 to M7. ACTH, adrenocorticotropic hormone; EPO, erythropoietin; FL, flt-3 ligand; IL-3, interleukin-3; IL-6, interleukin-6; α-MSH, α-melanocyte stimulating hormone; SCF, stem cell factor; TPO, thrombopoietin. (**B**) May-Grunwald-Giemsa staining of control erythroblasts at day M0–5 day. Cells differentiated to Pro-E stage at day M0 and Baso-E stage at day M3. Arrows, Poly-E; Arrow heads, Orho-E. *, Reticulocyte. Bar 10 μm. (**C**) Conventional RT-PCR for MCRs during erythroblasts differentiation between M0 and M6 day.

CD34^+^ hematopoietic progenitor cells (HPCs) derived from human umbilical cord blood (purity, >90%, Lonza) [34–36] were used in the experiments. In the first passage (for expansion; E0–E7 in Fig 1A), CD34^+^ cells at 1 × 10^5^ cells/ml were cultured in 20 ml of hematopoietic progenitor growth medium (HPGM, Lonza) supplemented with 25 ng/ml recombinant human stem cell factor (SCF, PeproTech), 50 ng/ml human thrombopoietin (Sigma-Aldrich), and 50 ng/ml Flt3 ligand (PeproTech) for 7 days [37]. Once the cells had proliferated to 2 × 10^6^ cells/ml, stock cultures were prepared at 1 × 10^6^ cells/bottle in liquid nitrogen. In the second passage (for differentiation; D0–D3 in Fig 1A), cell stocks were thawed and cultured at 2 × 10^5^ cells/ml in HPGM supplemented with 3 U/ml human EPO (Kyowa Hakko Kirin), 25 ng/ml SCF, 10 ng/ml recombinant human IL-3 (PeproTech), and 10 ng/ml recombinant human IL-6 (R&D Systems) for 3 days (Fig 1A). In the third passage (for maturation; M0–M3 in Fig 1A), cells at 1 × 10^5^ cells/ml were cultured in HPGM supplemented with EPO. On the third day of maturation (M3), an approximately 4-fold dilution of the cells was cultured.

This study was carried out in strict accordance with the recommendations in the Declaration of Helsinki. All procedures involving experimental human cells were performed in accordance with the guidelines provided by the Ethics Committee of Kanazawa Medical University (Permit Number: 60).

### May–Grunwald Giemsa stain

Cultured cells were spun onto slides using a Cytospin 4 (Thermo Scientific). The cells were stained with May–Grunwald–Giemsa solution. The cells were then observed under a LSM 710 Laser Scanning Microscope with AxioCam (Carl Zeiss). The differentiation stage of erythroblasts was determined and the proportion of erythroblasts in different stages was calculated by counting 100 cells/slide.

### RNA extraction and reverse transcription (RT) PCR analysis

Erythrocyes harvested on M0–6 were first briefly homogenized in lysis solution to increase yield efficiency. Total cellular RNA was extracted using RNAeasy Kit (Qiagen) according to the manufacturer’s protocol. Equal amount of RNA samples were reverse-transcribed by using Superscript VILO cDNA synthesis kit (Invitrogen). PCR amplification of cDNA was carried out using Takara Ex Taq Hot Start Version (Takara Bio, Inc., Japan) and the gene-specific primer sets as follows: MC1R (forward, 5’- actccaggcaggacttctca -3’; reverse, 5’- agtgcccagtctgagcctta -3’) (443 bp), MC2R (forward, 5’- ggcaaagacttgctttcctg -3’; reverse, 5’- cccacatgggaactaaatgg -3’) (467 bp), MC3R (forward, 5’-ctgctggaaaacatcctggt -3’; reverse, 5’-acggtcatgatgctgtggta-3’) (317 bp), MC4R (forward, 5’- tttactcacagcaggcatgg -3’; reverse, 5’- atccatttgaaacgctcacc -3’) (451 bp), MC5R (forward, 5’- ctgggagaccatcaccatct -3’; reverse, 5’- ggaggaacagcatagcgaag -3’) (332 bp). Glyceraldehyde-3-phosphate dehydrogenase (GAPDH, forward, 5’- gtggatggcccctccgggaa -3’; reverse, 5’-ggcagggactccccagcagt -3’) (520 bp). PCR was carried out for 35 cycles of amplification, consisting of 98°C for 10 seconds, 58°C for 30 seconds, and 72°C for 60 second. PCR products were electrophoresed in 1% agarose gel, stained with ethidium bromide, and visualized by ultraviolet transillumination.

For quantitative RT-PCR (qRT-PCR), first-strand synthesis was performed using the Superscript VILO cDNA synthesis kit (Invitrogen). qRT-PCR was performed using cDNA templates with Premix Ex Taq (Perfect Real Time; Takara Bio). TaqMan 18S rRNA (ABI) was used as an endogenous control. TaqMan probes for human MC5R (Hs990431_s1) were used. Reactions were carried out in triplicate using the ABI 7900HT Fast Real-Time PCR System.

### Enucleation percentage

ACTH39, ACTH24, andα-MSH (M4135) were purchased from Sigma-Aldrich. Melanocortins were dissolved in phosphate-buffered saline with 0.1% bovine serum albumin (BSA, ≥99%, A0281; Sigma-Aldrich).

The enucleation ratio is determined by microscopy-based cell counting after treatment with ACTH39, ACTH24 orα-MSH. Cells were transferred to a glass-bottom dish (Greiner Bio-One) and fixed with 10% (v/v) formalin. Bright-field and fluorescent images of cells stained with Hoechst33342 (2 μg/ml, Molecular Probes) were captured by epifluorescence microscopy from ten independent areas according to the systematic and random sampling method (IX-FLA, Olympus). The number of enucleating cells and enucleated cells were counted as enucleated RBCs. The enucleation ratio was then calculated as the percentage of the number of enucleated cells over the total number of cells [Enucleation ratio = (enucleating cells + enucleated cells)/total cells (300 cells)].

### Melanocortin subtraction [ACTH(−) and α-MSH(−) medium]

Fifty microliters of Dynabeads protein G (Invitrogen) were incubated with 10 μg of anti-ACTH mAb (an Ab against ACTH24; Santa Cruz) or 1 μl of anti-α-MSH serum (Millipore) for 10 min at room temperature (RT) and subsequently incubated with StartingBlock blocking buffer (Thermo Scientific) for 10 min at RT. Ab-conjugated beads were incubated in HPGM for 10 min at RT and the beads were removed using magnets. Control IgG medium was prepared using normal mouse IgG (Dako) and goat serum.

### Inhibitors of MCRs

The neutralizing antibodies (nAbs) for MCRs were rabbit anti-human MC1R polyclonal Ab (pAb) (1.25 μg/ml, ab97321; Abcam), rabbit anti-human MC2R pAb (2.5 μg/ml; Millipore), rabbit anti-human MC3R pAb (5 μg/ml, ab21231; Abcam), rabbit anti-human MC4R pAb (5 μg/ml, ab75506; Abcam), goat anti-human MC5R pAb (5 μg/ml, ab92287; Abcam), rabbit anti humanα-MSH pAb (30 μg/ml), and mouse anti-human ACTH monoclonal Ab (mAb) (15 μg/ml, B427; Santa Cruz Biotechnology). Normal rabbit IgG (ab37415; Abcam), goat IgG (ab37373; Abcam), and mouse IgG (Millipore) were used as negative controls. HS 024 (Tocris Bioscience) was used as an antagonist of MC4R, and agouti (1–40)-amide human (Phoenix Pharmaceuticals) was used as an antagonist of MC3R and MC4R.

### Flow cytometric analyses

CD71 and CD235a positive cells were detected with FITC-conjugated CD71 mAb (1:100, Becton, Dickinson) and APC-conjugated CD235a (glycophorin A) mAb (1:500, Beckton Dickinson). Analyses of MCR expression levels were undertaken according to a specific procedure. In brief, cells were fixed with 4% paraformaldehyde and blocked with Human TruStain FcX (Fc Receptor Blocking Solution, Bio-legend, CA, USA) for 15 min. The cells were incubated with goat anti-human MC5R pAb (25 μg/ml; Abcam) in Tris-HCl buffer with 0.25% Triton X-100 for 1 h and then incubated with secondary Abs [Alexa Fluor 488-labeled donkey anti-rabbit IgG (Molecular Probes) or Alexa Fluor 488-labeled donkey anti-goat IgG] for 30 min at RT.

### Western blotting

Cell lysates (1 × 10^5^ cells) were separated on a polyacrylamide gel using the Laemmli discontinuous buffer system. Proteins (ERK, p-stat5, and AKT, 1 × 10^5^ cells/ lane; p-ERK, STAT5, p-AKT, MLC2, and p-MLC2, 4 × 10^5^ cells/ lane) were loaded onto a 5–20% or 10–20% SDS-PAGE gel and transferred onto a PVDF membrane. The membranes were blocked with 5% BSA (Sigma-Aldrich) in Tris-buffered saline (TBS) with 0.1% Tween-20 (TTBS) and incubated overnight at 4°C with the following Abs in 5% BSA-TTBS: phospho-p44/42 MAPK (Erk1/2) (Thr202/Tyr204) rabbit mAb (1:1000), p44/42 MAPK (Erk1/2) rabbit mAb (1:1000), phospho-Stat5 (Tyr694) rabbit mAb (1:1000), Stat5a mouse mAb (1:1000), phospho-Akt (Ser473) rabbit mAb (1:1000), Akt rabbit mAb (1:1000), MLC2 rabbit mAb (1:1000), and phospho-MLC2 (Thr18/Ser19) rabbit mAb (1:1000). All Abs were obtained from Cell Signaling Technology. After thorough washing, the membranes were incubated with biotinylated anti-rabbit IgG (1:2500) (Zymed Laboratories) or biotinylated anti-mouse IgG (1:2500) (Zymed) for 1 h at RT and reacted with extra-avidin peroxidase (Sigma-Aldrich) (1:2000) for 15 min at RT. The avidin–peroxidase conjugate was detected on the basis of enhanced chemiluminescence (Luminata Forte Western HRP Substrate; Millipore) using the Lumino image analyzer LAS-4000 (Fuji Film).

### Immunostaining

Erythroblasts were attached on the slide glasses coated with gelatine prior to fixation. The cells were fixed in 4% (*v/v*) paraformaldehyde and blocked with 10% normal donkey serum in StartingBlock (Thermo Scientific) for 30 min at RT. The cells were incubated with goat anti-MC5R pAb (25 μg/ml; Abcam) in TBS with 0.25% Triton X-100 overnight at 4°C before incubating in Alexa Fluor 488-labeled donkey anti-goat IgG for 30 min at RT. Alexa Fluor 594-labeled phalloidin (Molecular Probes) and APC-conjugated CD235a (glycophorin A) mab were allowed to react for 20 min at RT. Phospho-Akt (Ser473) rabbit mAb (1:50) and phospho-MLC2 rabbit mAb (1:200) were used. Hoechst33342 was used for nuclear staining. The cells were then observed under an LSM 710 Laser Scanning Microscope (Carl Zeiss).

### ELISA for ACTH

Measurements of the ACTH39 level in HPGM and albuminate (Nihon Pharmaceutical) were performed using electrochemiluminescence immunoassay (SRL Inc.). ACTH24/39 was detected by sandwich ELISA, a method that we have established previously for the measurement of ACTH1–24 and ACTH1–39 fragments [31]. ACTH was captured using anti-mouse ACTH mAb (B427) (Santa Cruz Biotechnology) and detected using anti-ACTH pAb (Chemicon) labeled with biotin. mAb was raised against amino acids 1–24 of human ACTH. An ACTH fragment (ACTH 1–24 of human/rat, Sigma–Aldrich) was used as a standard. All plates were blocked with Starting Block (Thermo Scientific). Biotin was reacted with an extra-avidin–peroxidase conjugate (Sigma–Aldrich). Absorbance signals were generated using TMB substrate solution (Thermo Scientific) and measured using a 2104 EnVision plate reader (Perkin Elmer).

### Extracellular fluid in mouse bone marrow

C57BL/6J male mice aged 24–32 weeks (Japan SLC, Shizuoka, Japan) were anesthetized by injection with pentobarbital (30 mg/kg, i.p.). Bone marrows were harvested (almost 12 μl per mouse) from the femurs of mice and mixed with 5 μg/ml FITC-dextran (Sigma-Aldrich) in PBS. After centrifugation, FITC concentration in the supernatant was measured by EnVision (Perkin Elmer) and the dilution ratio was calculated. The ACTH level in the supernatant was measured by ELISA.

This study was carried out in strict accordance with the recommendations in the Guide for the Care and Use of Laboratory Animals of Kanazawa Medical University. The protocol was approved by the Committee on the Ethics of Animal Experiments of the Kanazawa Medical University (Permit Number: H20 No20). All surgery was performed under sodium pentobarbital anesthesia, and all efforts were made to minimize suffering.

### Statistical analyses

All measurements are given as the mean of three experiments. Each analysis was performed using an analysis of variance (ANOVA) and a *post hoc* test (Fisher’s protected least significant difference). *P* < 0.05 was considered significant. Values are presented as mean ± standard error or standard deviation.

## Results

### MCRs expression in human erythroblasts

The culture protocol modified from Lonza was altered to allow CD34^+^ human HPCs to differentiate into erythroid cells (Fig 1A) [38]. A total of 97.0% erythroblasts were glycophorin A^+^ (GPA^+^)/CD71^+^ at M3 (S1 Fig). The cells were pro-erythroblasts at M0 and then started to enucleate at M5 (Fig 1B). We detected whether MCRs expressed in human erythroblasts, because we reported that MC2R and MC5R expressed in rodents [31]. *MC1-5R*s mRNA expressed during erythroblast differentiation between M0 and M6 day (Fig 1C).

### ACTH promotes erythroblast differentiation

**Fig 2:**
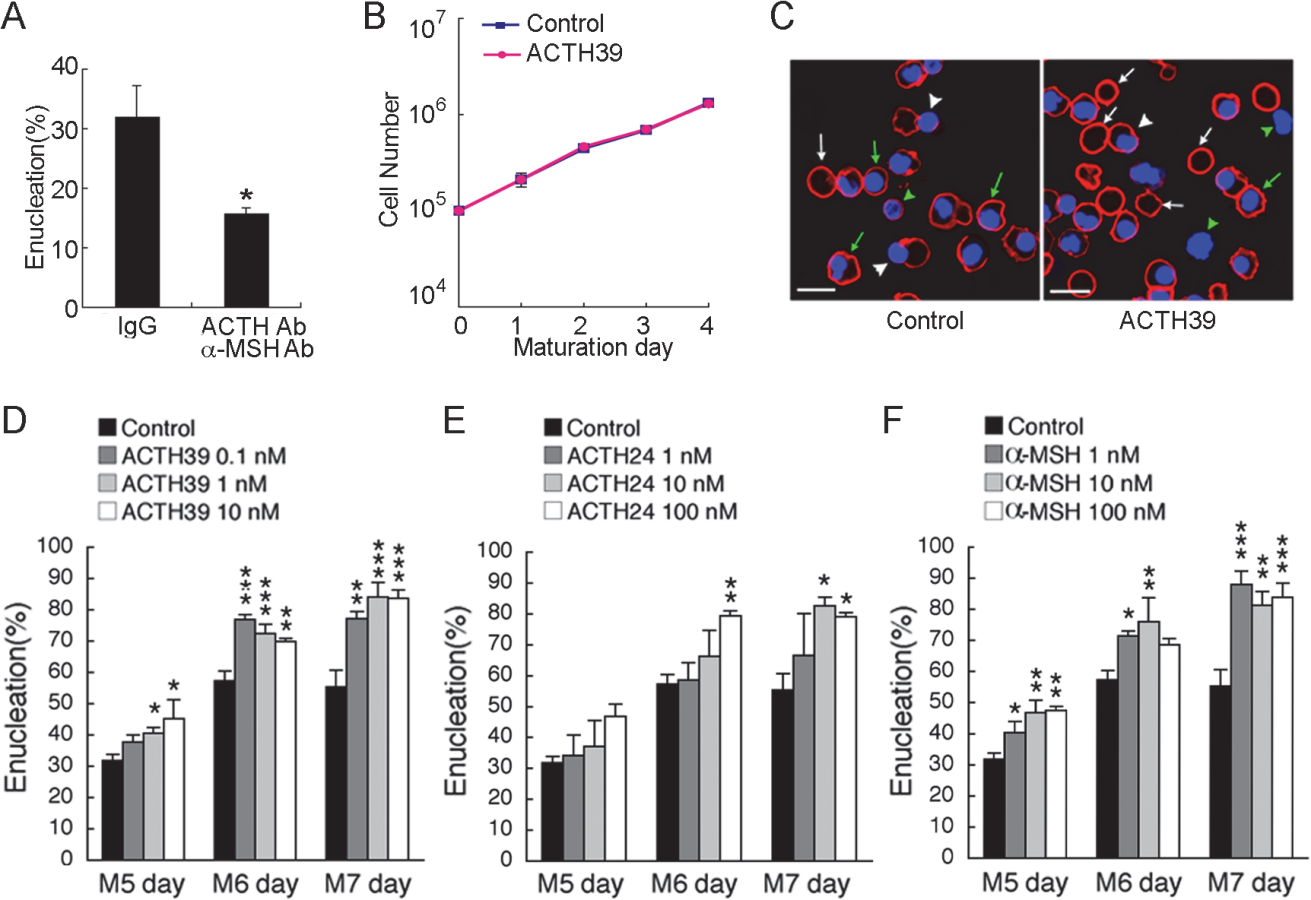
ACTH enhances enucleation of erythroblasts derived from human HPCs. **(A)** The neutralization of melanocortins by addition of the neutralising antibodies (nAbs) for ACTH and α-MSH into culture media on the M0 day and the M3 day. The enucleation rate on the M5 day was measured. *, P < 0.01. n = 3; error bars, s.e.m. (**B**) Cell number between M0 and M4 day. ACTH39 has no significant effect on the proliferation of erythroblasts. n = 3; error bars, s.e.m. **(C)** The representative images of definitive erythroid differentiation induced by melanocortins on the M5 day. The glycophorin A (GPA)-positive cells without nuclei are increased in number by treatment with 10 nM ACTH39. Control, vehicle (0.1% BSA); ACTH39, 10 nM ACTH39; GPA, APC (red); nuclei, Hoechst33342 (blue). White arrows, enucleated cells; White arrowheads, enucleating cells; Green arrows, normal erythroblasts; Green arrowheads, excreted nuclei. Bar, 20 μm. The enucleation rates in definitive erythroid cells by treatments with **(D)** ACTH39, **(E)** ACTH24, and **(F)** α-MSH between the M5 and M7 days. All melanocortins showed effects on the induction of enucleation by erythroblasts. *, P < 0.01; **, P < 0.001; ***, P < 0.0001, each point versus control in the same day. n = 3; error bars, s.e.m.

Under conventional culture conditions using HPGM, erythroblasts can also differentiate into terminal erythroid cells without additional melanocortin treatments. We confirmed that the contamination level of ACTH39 in HPGM was 0.8 pM, and the contamination level of ACTH24/39 (including the 1–24 and 1–39 fragments of ACTH) was 249.3 pM (S1 Table). Moreover, albuminate, which is albumin extracted from human plasma, contained ACTH (S1 Table). When we removed ACTH and α-MSH (ACTH1–13 fragment) from HPGM to decrease ACTH24/39 concentration to 12.9 pM (data not shown) [ACTH(−) andα-MSH(−)], the enucleation ratio with ACTH(−) andα-MSH(−) was lower than that of the control treated with control IgG for 7 days (Fig 2A). Addition of the ACTH1–39 fragment (ACTH39) beginning at M0 did not affect proliferation (Fig 2B). The enucleation ratio including enucleated (white arrows in Fig 2C) and enucleating RBCs (white arrowheads in Fig 2C) [19], increased by the addition of ACTH39, ACTH24 or α-MSH (control: 55.4%, 10 nM ACTH39: 83.7%, 100 nM ACTH24: 79.1%, 1 nMα-MSH: 88.9% at M7), indicating that most erythroblasts had the potential for enucleation (Fig 2D–2F). ACTH39 and its fragments were contained in conditioned medium for erythropoiesis; however, the concentration of ACTH fragments (or derivatives) was not sufficiently high to promote erythroid differentiation. Therefore, differentiation and enucleation were accelerated by melanocortin addition (Fig 2D–2F), indicating that melanocortins are important factors for terminal erythroblast differentiation. In fact, as a humoral factor, ACTH24/39 concentration in the extracellular fluid of adult mouse bone marrow was higher than that in the peripheral serum (bone marrow, 383.2 ± 53.2 nM; serum, 109.7 ± 3.5 nM, *P <* 0.01) (S2 Fig).

### MC1R, MC2R, and MC5R contribute to erythroblast differentiation

**Fig 3:**
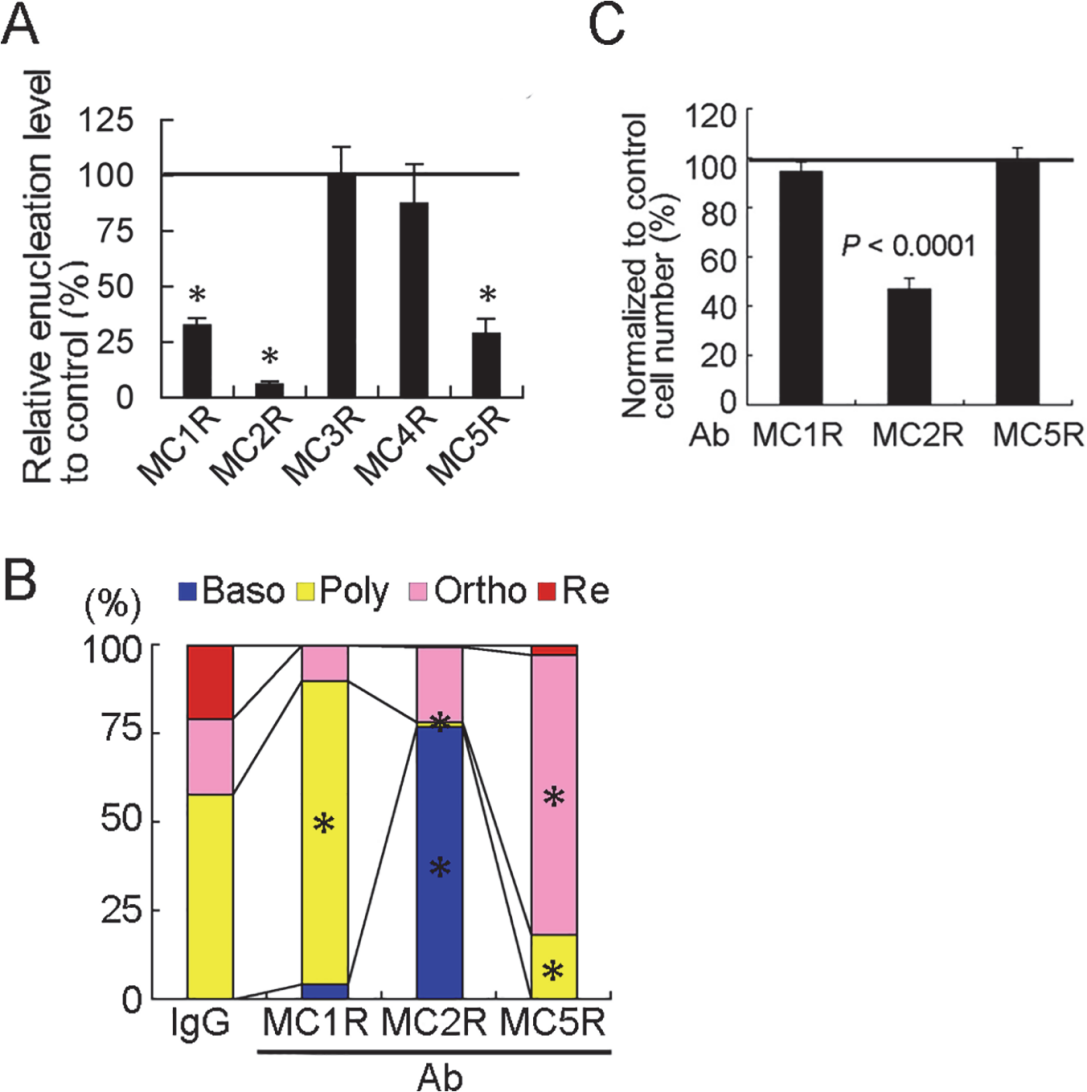
Blocking of MC1R, MC2R or MC5R causes a decrease in the enucleation ratio of erythrocytes. **(A)** MC1–5R nAb were added to the culture medium on M0 and M3, and the enucleation ratio was analyzed at M7. The enucleation ratio was decreased in the erythroblasts treated with anti-MC1R nAb, anti-MC2R nAb or anti-MC5R nAb. *, *P <* 0.0001, each point versus control. n = 3; error bars, s.e.m. (**B**) The number of erythroblasts at the polychromatic erythroblast stage, the basophilic stage and the orthochromatic stage increase after treatment with the anti-MC1R nAb, anti-MC2R nAb and anti-MC5R nAb, respectively. Baso, basophilic erythroblasts; Poly, polychromatic erythroblasts; Ortho, orthochromatic erythroblasts; Re, reticulocyte. *, *P* < 0.001. Values are compared with cells treated with control IgG. **(C**) The effects of MC1R, MC2R or MC5R nAbs on erythroblast proliferation at M5 day. Cell number is significantly decreased after treatment with anti-MC2R nAb for 5 days; however, treatment with anti-MC1R nAb or anti-MC5R nAb does not have a significant effect (n = 3, ANOVA). Error bars, s.e.m.

Next, we tested whether the enucleation ratio was inhibited by treatment with nAbs for MC1–5R. The enucleation ratio of erythroblasts treated with anti-MC1R,-MC2R, and-MC5R nAbs decreased significantly compared with that of erythroblasts treated with normal IgG (control) (Fig 3A). Anti-MC3R nAb or anti-MC4R Ab could not decrease the enucleation ratio (Fig 3A). Furthermore, neither agouti, an antagonist of MC3R and MC4R [39,40], nor HS024, an antagonist of MC3R [41], decrease enucleation ratio (S3 Fig). The MC1R-blocked cells accumulated at the polychromatic erythroblast stage at M5, and the MC2R-blocked cells failed to differentiate further, resulting in cell accumulation at the basophilic erythroblast stage (Fig 3B). The MC5R-blocked cells accumulated at the orthochromatic erythroblast stage at M5 (Fig 3B). The enucleation ratio of erythroblasts treated with anti-MC2R nAb was the lowest (Fig 3A), suggesting that MC2R contributes to the proliferation as well as differentiation of erythroblasts (Fig 3C).

### ERK, STAT5, and AKT phosphorylation are regulated by MC2R-, MC1R-, and MC5R-mediated signals

**Fig 4:**
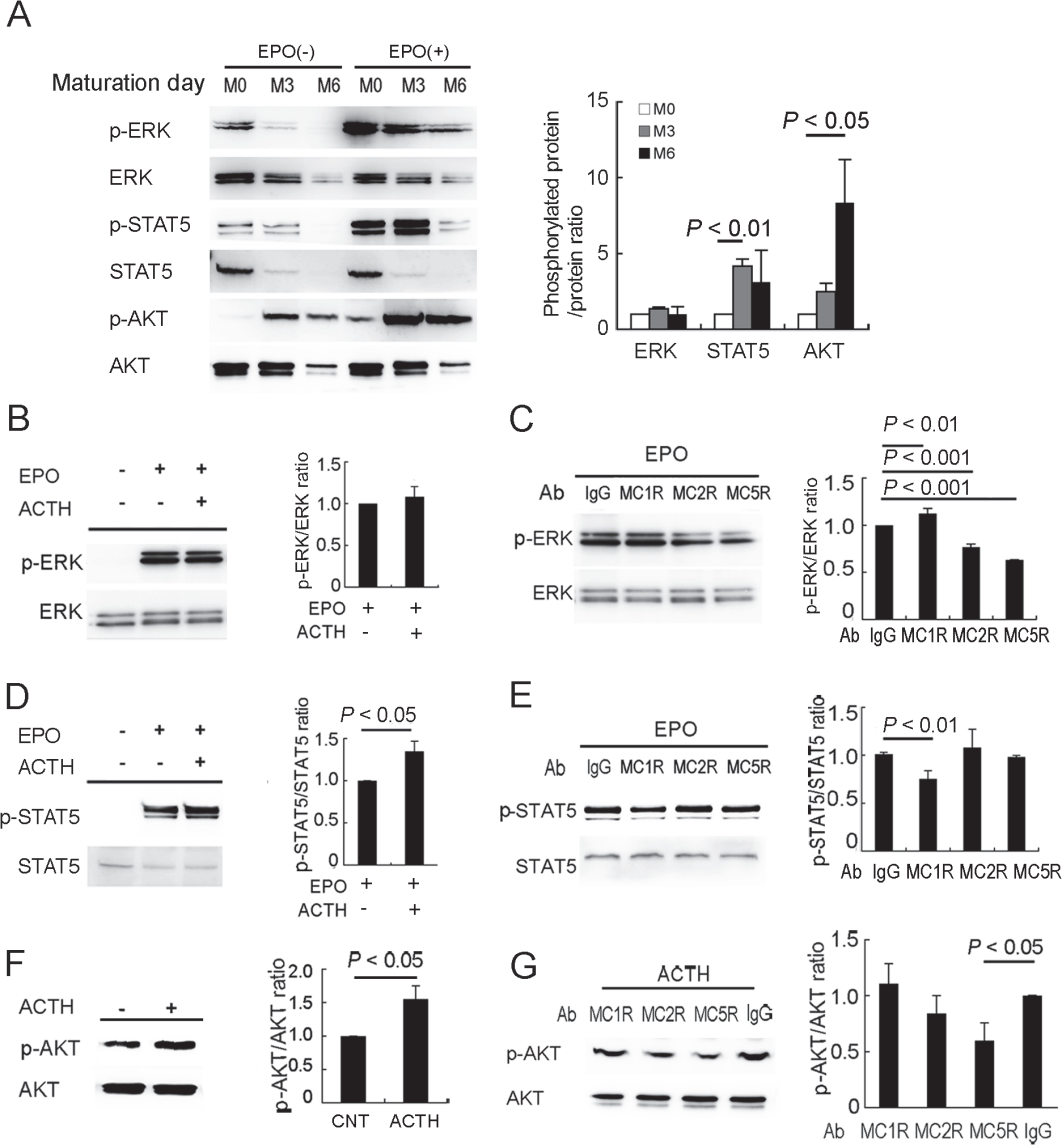
MCRs differentially regulate EPO-induced phosphorylation of ERK, STAT5 and AKT in erythroblasts during differentiation. **(A)** Phosphorylation of ERK, STAT and AKT in erythroblasts. Erythroblasts at M0, M3 and M6 were starved for 3 h in HPGM without cofactors and subsequently incubated for 15 min with or without EPO. (**B**)**–**(**E**), Synergistic effects on EPO downstream signaling of ACTH, and the signal inhibition by nAbs. After starvation with HPGM without cofactors for 3 h, the cells were incubated for 15 min with nAbs and reacted with EPO for 15 min. EPO-induced phosphorylation of ERK is not altered by the addition of 0.1 nM ACTH39 to erythroblasts at M0. n = 3; error bars, s.e.m **(B)**. The phosphorylation of ERK is decreased by nAbs of MC2R and MC5R **(C)**. ACTH39 enhances EPO-induced phosphorylation of STAT5 in erythroblasts at M3 **(D)**. Phosphorylation of STAT5 is inhibited by nAbs of MC1R **(E)**. **(F)** and **(G),** AKT phosphorylation by ACTH and, the signal inhibition by nAbs. Treatment with ACTH39 for 15 min without starvation causes phosphorylation of AKT in erythroblasts at M6 **(F)**. The phosphorylation of AKT is inhibited by MC5R-nAb **(G)**. EPO, 3 U/ml EPO; ACTH, 0.1 nM ACTH39. n = 3; error bars, s.e.m. IgG, 10 μg/ml normal IgG; MC1R, 10 μg/ml anti-MC1R nAb; MC2R, 5 μg/ml anti-MC2R nAb; MC5R, 10 μg/ml anti-MC5R nAb. n = 3; error bars, s.e.sm.

To clarify whether the melanocortin system modulates these signals in erythroblasts, we analyzed the phosphorylation status of ERK, STAT5, and AKT in erythroblasts. First, we performed immunoblot analyses to examine phosphorylation from M0 to M6. The amount of phosphorylated ERK (p-ERK) was the highest at M0, although the p-ERK/ERK ratio did not change between M0 and M6. The p-STAT5/STAT5 ratio peaked at M3, and the p-AKT/AKT ratio increased until M6 with EPO treatment (Fig 4A). Although EPO-induced p-ERK was not enhanced by treatment of erythroblasts with ACTH39 at M0 (Fig 4B), it was inhibited by nAbs for MC2R and MC5R (Fig 4C). On the other hand,s, when the erythroblasts were starved with Iscove’s modified Dulbecco’s medium (IMDM), which does not contain ACTH, EPO-induced ERK phosphorylation was clearly enhanced by ACTH addition (S4 Fig). These findings are consistent with our result indicating that proliferation was not altered by ACTH39 treatment (Fig 2B), erythroblast proliferation was inhibited by anti-MC2R nAb (Fig 3C) and erythroblast differentiation was arrested at the baso-E stage by nAb (Fig 3B). Signals transduced *via* MC2R might be major modulators of the ERK signal. EPO-induced STAT5 phosphorylation was enhanced by ACTH39 addition (Fig 4D) and was inhibited by anti-MC1R nAb (Fig 4E). In our study, MC1R-blocked erythroblasts accumulated at the poly-E stage (Fig 3B) at which erythroblasts efficiently produce hemoglobin, suggesting that STAT5 regulates IRP-2 expression [13]. At M6, AKT was phosphorylated by ACTH addition without starvation (Fig 4F), and activation was inhibited by anti-MC5R nAb (Fig 4G). P-AKT was co-expressed strongly with MC5R (S5 Fig). These results suggest that erythroblast differentiation is regulated in a stage-specific manner by MC2R, MC1R, and MC5R.

### Enucleation is regulated by a MC5R-mediated signal

**Fig 5:**
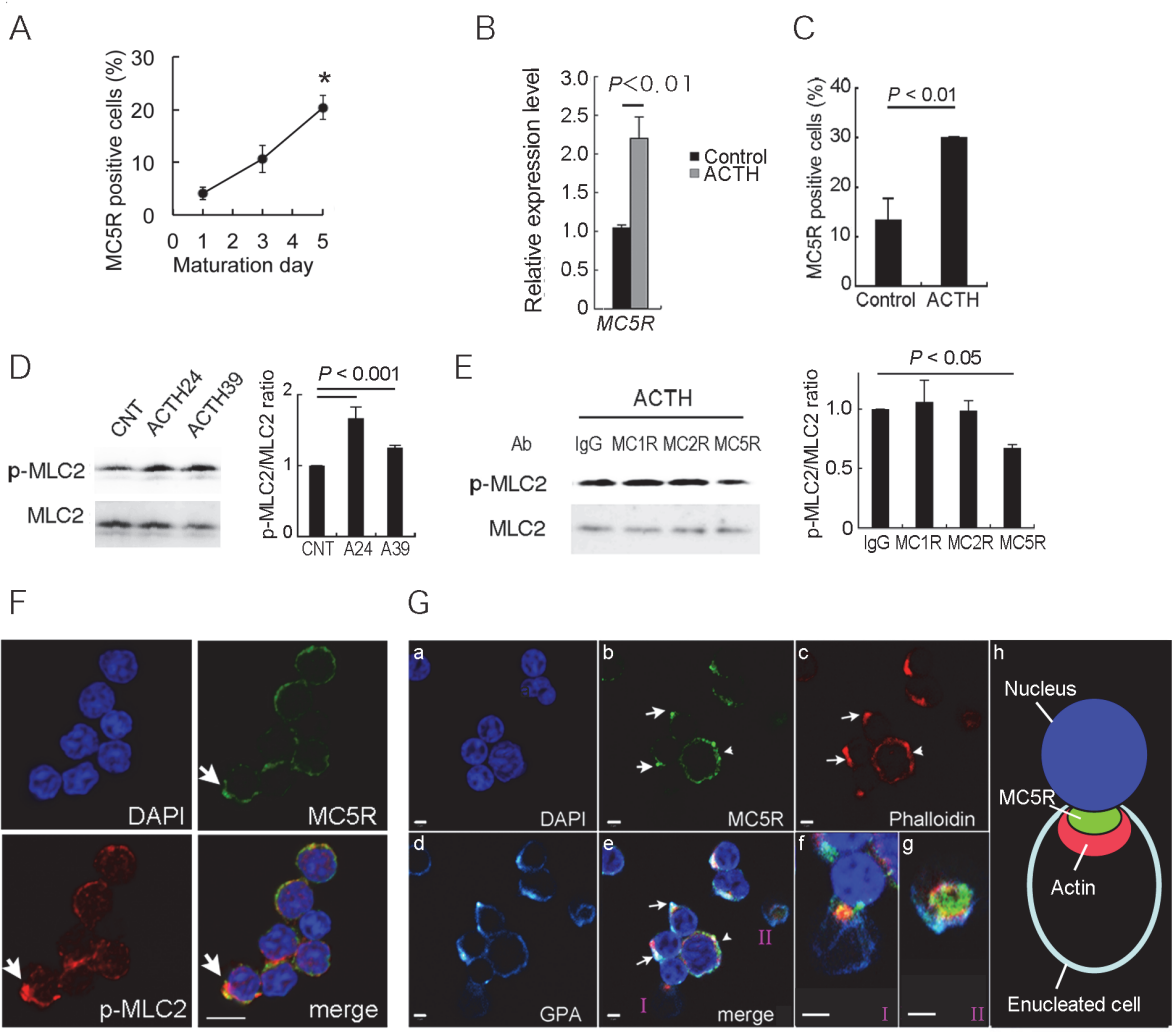
The MC5R signal is required for enucleation. **(A)** MC5R-positive cell ratio during maturation. The percentage of MC5R-positive cells is increased between M1 and M5 day by flow cytometric analysis. n = 3; error bars, s.e.m. (**B)** and **(C**), Expression of MC5R increased by ACTH treatment. **(B)** Changes in *MC5R*s mRNA expression are induced by treatment with 0.1 nM ACTH39 at M4. The expression levels *MC5R* is significantly elevated by ACTH39 treatment. The *MCR* expression levels in the ACTH39-treated groups represent the levels relative to the controls. n = 3; error bars, s.e.m. (**C**) The ratio of MC5R-positive cells increased by ACTH treatment. n = 3; error bars, s.e.m. **(D)**The addition of 0.1 nM ACTH39 (A39) and 5 nM ACTH24 (A24) causes phosphorylation of MLC2 in erythroblasts at M6 without starvation. **(E)** ACTH39-dependent phosphorylation is inhibited by anti-MC5R nAb. n = 3; error bars, s.e.m. **(F)** and **(G),** Confocal laser scanning microscopic images of enucleating and enucleated cells at M6. **(F)** Phosphorylated MLC2 co-localizes with MC5R at the plasma membrane (white arrows). Bar, 5 μm. **(G)** MC5R is localized on the membrane (arrowheads, in panel b, c, e) with actin. White arrows, MC5R co-localizes with an actin-accumulating locus (panel b, c, e). Panel f or g is an enlarged image from I or II of panel e, respectively. Panel f, MC5R co-localizes with the contractile actomyosin ring in an enucleating cell. Panel g showed cross-section surface of contractile ring. Panel h (schema) is obtained schematizes f and g. Panel a, Nuclei, DAPI (blue); Panel b, MC5R, Alexa488 (green); Panel c, F-actin, Phalloidin-Alexa596 (red); Panel d, GPA, APC (light blue). Bars, 2 μm.

When flow cytometric analyses revealed an alteration of the MC5R^+^ cell ratio from M1 to M5, the MC5R^+^ cell ratio increased (M1: 4.1%, M3: 10.6%, M5: 20.4%) (Fig 5A). The result is consistent with *MC5R* mRNA tended to increase between M0 and M6 (Fig 1C). We analyzed the effect of the MC5R signal on enucleation. *MC5R* expression increased significantly with ACTH39 at M4 (Fig 5B), and the ratio of MC5R^+^ cells increased to 30.1% (control: 13.5%) (Fig 5C). Phosphorylation of MLC2 and the assembly into a contractile actomyosin ring was increased by the addition of ACTH (ACTH24 and ACTH39) (Fig 5D) at M6, and this phosphorylation was inhibited by nAbs for MC5R (Fig 5E). Phosphorylated-MLC2 co-localized strongly with MC5R at M6 (Fig 5F). Of note, MC5R co-localized peripherally with F-actin at the plasma membrane (arrowheads in panel b, c, e of Fig 5G). Subsequently, F-actin was integrated into an actin ring on which MC5R accumulated (arrows in panel b, c, e of Fig 5G) in enucleating erythroblasts. Contractile ring was formed in an enucleating cells (panel f in Fig 5G) and MC5R was also localized to the periphery of the contractile ring (panel g and h in Fig 5G), suggesting that the MC5R-mediated signal is part of an upstream signaling pathway for enucleation, which is consistent with the finding that MC5R-blocked cells accumulated at the ortho-E stage (Fig 3B).

## Discussion

The present study revealed that melanocortins play critical roles in inducing erythroid differentiation including enucleation. The effects of melanocortins are sequentially regulated by MC2R-, MC1R-, and MC5R-mediated signals, depending on the differentiation stages of erythroblasts. The erythroblasts mature to terminal erythroid cells in culture media with the addition of EPO and human plasma components [2,42]. ACTH is contained in extracts of serum, fetal bovine albumin, and human plasma albumin, which are all used in pre-formed hematopoietic culture medium as common additives without the intention of adding specific factors for erythroid maturation. The contamination of ACTH in the culture media (S1 Table) resulted in a small alteration of EPO receptor (EPOR) signaling with the addition of ACTH because it constitutively affected the phosphorylation of downstream signaling of EPOR as described below in the control. This is supported by the significant alteration of phosphorylation of ERK induced by ACTH starvation (S4 Fig). The effects of ACTH on EPO/EPOR signaling in erythropoiesis, phosphorylation of STAT5, MAPK p44/42 (ERK1/2), and PI3K-AKT, which are the major downstream signals of EPO/EPOR, were activated to participate in the cell proliferation or survival as described below. ACTH concentration in adult mouse bone marrow was higher than that in the peripheral serum (S2 Fig). In rat embryos, ACTH was maintained at high concentrations in the serum and secreted from the placenta [31]. Recently, it was reported that the placenta could be a site for the terminal differentiation of primitive erythroid cells in the first trimester in humans [33], which suggests that placental ACTH and α-MSH function as differentiation/maturation and enucleation factors in embryonic erythropoiesis.

In the present study, we revealed that all MCRs are differentially expressed in human erythroblasts depending on the differentiation stages. Differentiation from the baso-E to the poly-E stage is first regulated by MC2R. Then, the differentiation from the poly-E to the ortho-E stage is mediated by an MC1R signal. Finally, enucleation from orthochromatic erythroblasts is induced by a MC5R-mediated signal.

MC1R has been shown to be expressed in macrophages [28], lymphocytes [43] neutrophils [29], dendritic cells [44], microglia [45], and the skin [46]. The present study is the first to report expression in erythroblasts. MC1R activates cAMP and ERK1/ERK2 signaling in the skin [47], but there are no reports of STAT5 signaling in association with MCRs. STAT5 is required for the transcription of CD71, IRP-2, and Bcl-xL [13], suggesting that it regulates erythroblast maturation *via* cellular iron uptake and anti-apoptosis. MC1R-blocking caused the inhibition of STAT5 signaling and then differentiation was arrested at the poly-E stage. However, blocking of MC1R did not cause a decrease in the number of proliferating cells. Instead, of Epo-ERK signaling that regulates proliferation, the modulating effects of MC1R on EPO-inducing signaling may lead to habitat segregation in the regulation by STAT5 of erythroblast differentiation (Fig 4).

MC2R is expressed in the adrenal gland [48], skin [49], lymphocytes [43], and embryonic nucleated blood cells [31]. The disruption of MC2R led to neonatal lethality in three quarters of the mice [50], and the number of erythrocytes decreased in surviving neonates compared with the wild-type [51]. MC2R and MC5R regulated lipolysis *via* p-ERK1/p-ERK2 in rodents [52]. ERK has roles in erythropoietin-dependent erythroblast proliferation [7], and this phenomenon was supported by the present study. ERK expression and phosphorylation decreased notably with erythroblast differentiation (Fig 4A); therefore, MC2R expression might depend on medium change of culture at M0 and M3 (Fig 1C).

MC5R is known to be a ubiquitously expressed receptor in adults and embryos [43,45,53 **–** 56] and is particularly expressed in exocrine glands [57]. Interestingly, the MC5R-mediated signal is associated with polarization (AKT) and formation of the contractile actomyosin ring (MLC2 and F-actin), suggesting that MC5R regulates enucleation. In the present study, MC5R expression increase with erythroblast differentiation (Fig 5A). ACTH addition increased the expression of *MC5R* mRNA and MC5R protein in erythroblasts (Fig 5B and 5C), suggesting that additional ACTH promotes not only enucleation but also MC5R expression. MC5R contributed to enucleation, and MC5R accumulated at the front of the plasma membrane in close contact with the enucleating nuclei (Fig 5G). This finding suggested that MC5R is associated with AKT (Fig 4F and 4G) and that MLC2 and the complex induce polarization and enucleation of orthochromatic erythroblasts.

**Fig 6:**
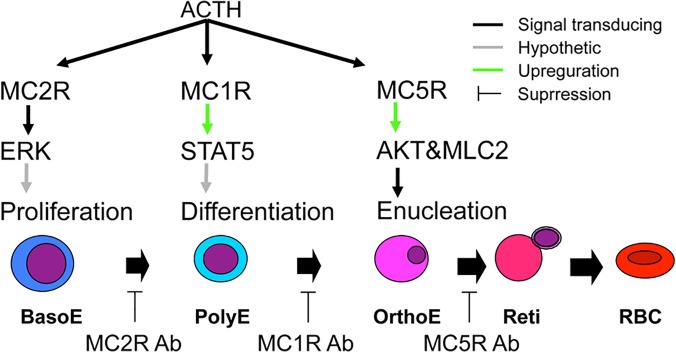
A hypothetical model of the MCR system that regulates erythropoiesis. MCR-induced activation of ERK, STAT5 and AKT are required for proliferation, differentiation, polarization and enucleation, respectively. We propose that MC1R and MC2R are involved in the modulation of responses to EPO. AKT and MLC2 are directly activated by an ACTH signal *via* MC5R. Baso-E, basophilic erythroblasts; Poly-E, polychromatic erythroblasts; Ortho-E, orthochromatic erythroblasts; Reti reticulocyte; RBC, red blood cell.

It remains unclear how MC2R, MC1R, or MC5R modulates ERK, STAT5, or AKT phosphorylation, respectively. Although MC2R is expressed constitutively, MC2R accessory protein, which interacts with MC2R and promotes receptor trafficking cytoplasm to the cell surface [58], may be involved in the regulation of MC2R modulated-ERK signaling. EPO signals are also sequentially regulated by modulating signals from the melanocortin–MCR system (ERK is regulated by MC2R, STAT5 by MC1R, and AKT by MC5R, Fig 6).

The multiple signaling cascades of STAT5, ERK1/2, and PI3K-AKT via EPOR are activated in an orchestrated manner [59], which may be temporally regulated by MCRs. Thus, the present study strongly suggests that MC1R, 2R, and 5R sequential but provably overlap each other to participate in the major three EPO-dependent erythropoietic signaling axes, STAT5, ERK1/2, and PI-3K-AKT, essential for proliferation, differentiation, and polarization during enucleation of erythroblasts.

The present study demonstrated a new framework for understanding the complexity of melanocortin-modulating EPO signals that are essential for erythropoiesis. This study also proposes the possible application of melanocortins in a new methodology for artificially inducing RBCs.

## Supporting Information

S1 Fig The population of CD71^+^/GPA^+^ erythroblasts at M1 and M3. For flow cytometric analyses, cells were stained with FITC-labeled mouse anti-human CD71 mAb and APC-labeled mouse anti-human CD235a mAb (glycophorin A:GPA). Cultured erythroblasts differentiate into GPA positive cells at M1. The signal intensity of CD71 decreases depending on the maturation stage of the erythroblasts.
(TIF)

S2 Fig The concentration of ACTH24/39 in mouse bone marrow. The concentration of extracellular ACTH in bone marrow was higher than that in serum (n = 3, ANOVA). BM, bone marrow. Error bars, s.e.m.
(TIF)

S3 Fig Effects of antagonists for MC3R and MC4R. Agouti (antagonist for MC3R and MC4R) or HS024 (antagonist for MC4R) were added to the culture medium on M0 and M3, and the enucleation ratio was analyzed at M7. The enucleation ratio did not alter by these antagonists.
(TIF)

S4 Fig The phosphorylation of ERK and STAT5. After starvation in IMDM medium, which does not contain ACTH, ACTH-induced p-ERK was detected by western blot. Furthermore, ACTH enhances the EPO-induced phosphorylation of ERK. ACTH, 0.1 nM ACTH39; EPO, 3 U/ml EPO.
(TIF)

S5 Fig Immunohistochemistry of MC5R and p-AKT. Localisation of p-AKT overlaps with that of MC5R in the periphery of cells. Arrow, P-AKT accumulated- cells expressed MC5R; Arrow head, p-AKT negative cells did not express MC5R. Bar, 5 μm.
(TIF)

S1 Table The concentration of ACTH39 and ACTH24/39 measured with ELISA. ACTH39 indicates the full-length of the ACTH peptide. ACTH24/39 indicates the concentration of the mixture of ACTH1-24 and ACTH1-39 fragments (See Materials and Methods).
(DOCX)
