# Supplementary figures and images for: Melanocortins Contribute to Sequential Differentiation and Enucleation of Human Erythroblasts via Melanocortin Receptors 1, 2 and 5
Source: PLoS One. 2015 Apr 10;10(4):e0123232. doi: 10.1371/journal.pone.0123232 (PMC4393082; doi:10.1371/journal.pone.0123232)

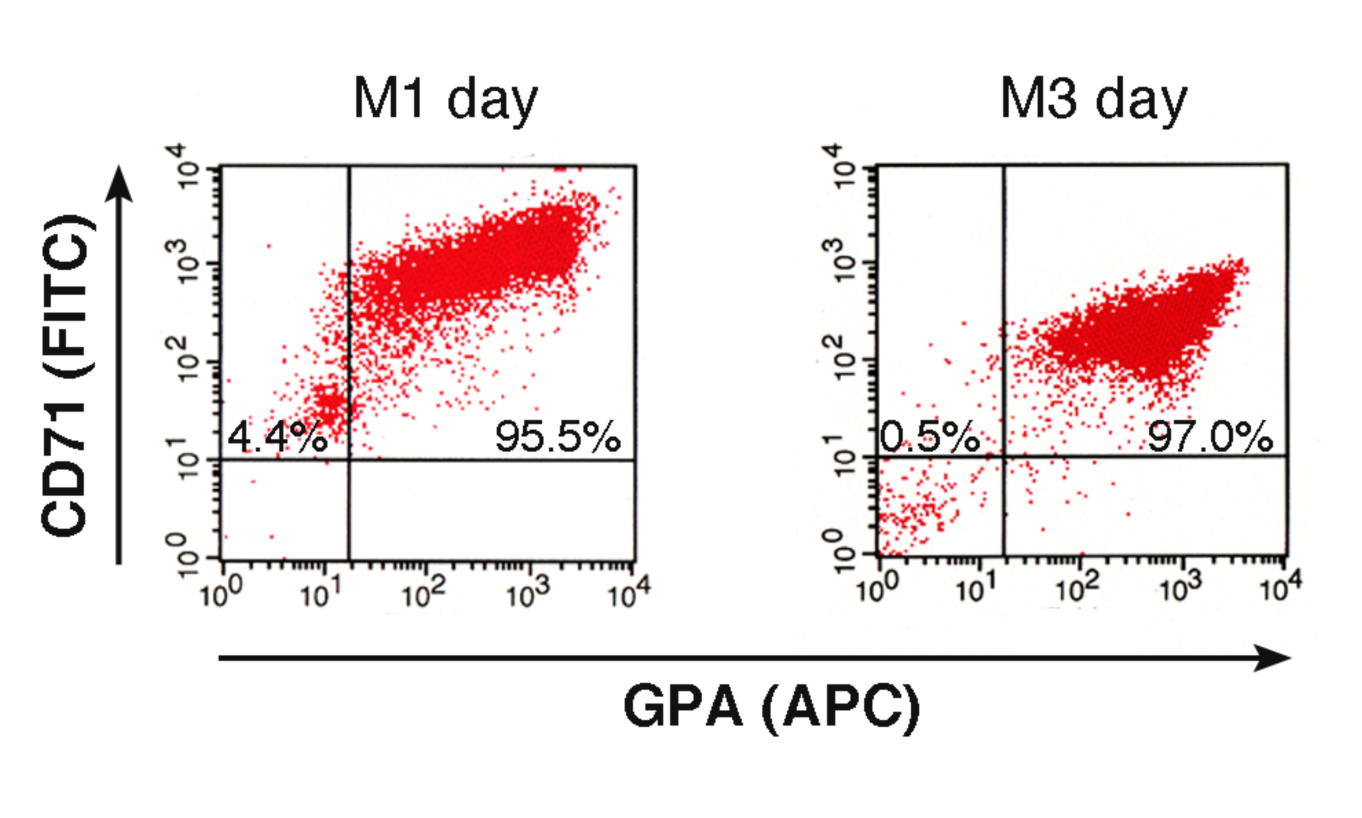

Supplement: S1 Fig — For flow cytometric analyses, cells were stained with FITC-labeled mouse anti-human CD71 mAb and APC-labeled mouse anti-human CD235a mAb (glycophorin A:GPA). Cultured erythroblasts differentiate into GPA positive cells at M1. The signal intensity of CD71 decreases depending on the maturation stage of the erythroblasts. (TIF) [file pone.0123232.s001.tif]

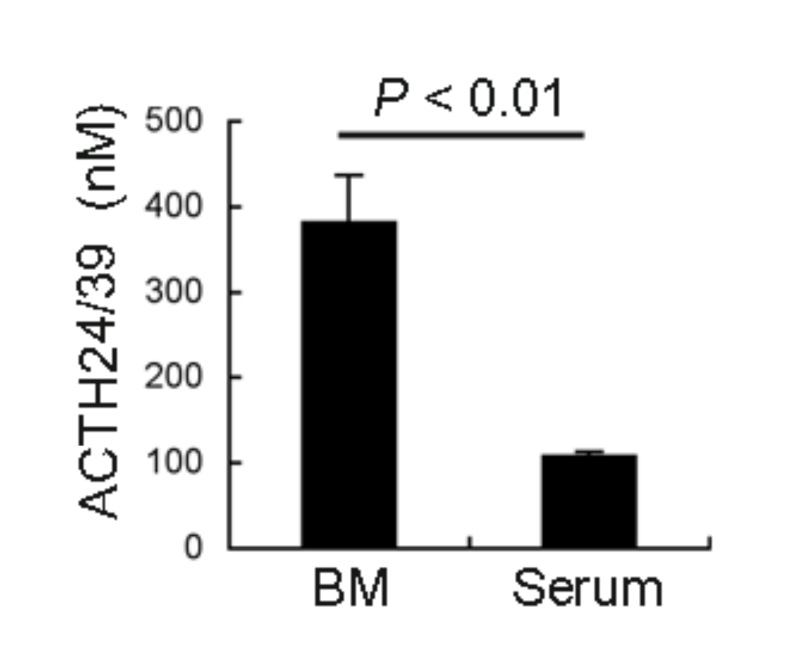

Supplement: S2 Fig — The concentration of extracellular ACTH in bone marrow was higher than that in serum (n = 3, ANOVA). BM, bone marrow. Error bars, s.e.m. (TIF) [file pone.0123232.s002.tif]

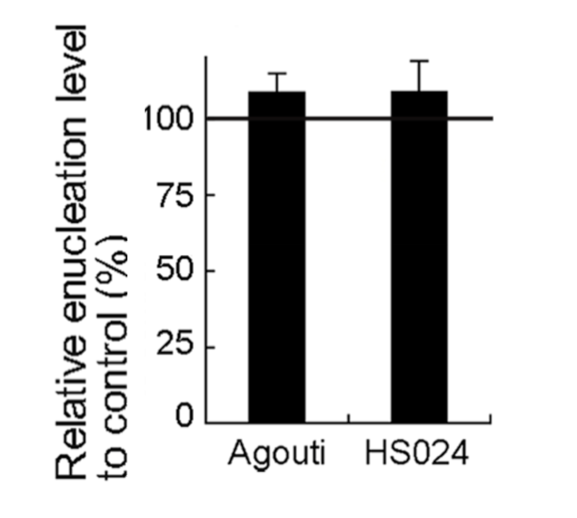

Supplement: S3 Fig — Agouti (antagonist for MC3R and MC4R) or HS024 (antagonist for MC4R) were added to the culture medium on M0 and M3, and the enucleation ratio was analyzed at M7. The enucleation ratio did not alter by these antagonists. (TIF) [file pone.0123232.s003.tif]

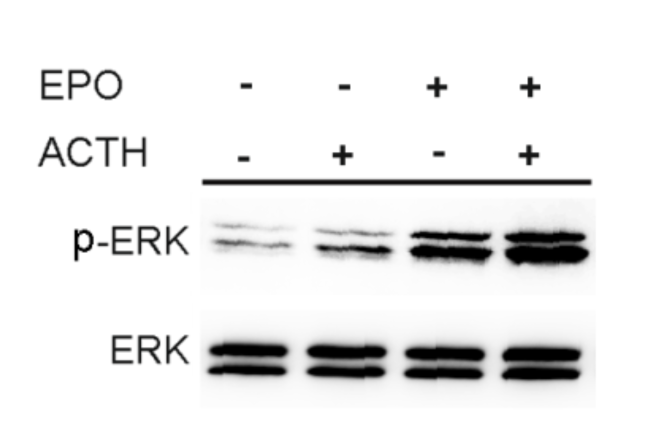

Supplement: S4 Fig — After starvation in IMDM medium, which does not contain ACTH, ACTH-induced p-ERK was detected by western blot. Furthermore, ACTH enhances the EPO-induced phosphorylation of ERK. ACTH, 0.1 nM ACTH39; EPO, 3 U/ml EPO. (TIF) [file pone.0123232.s004.tif]

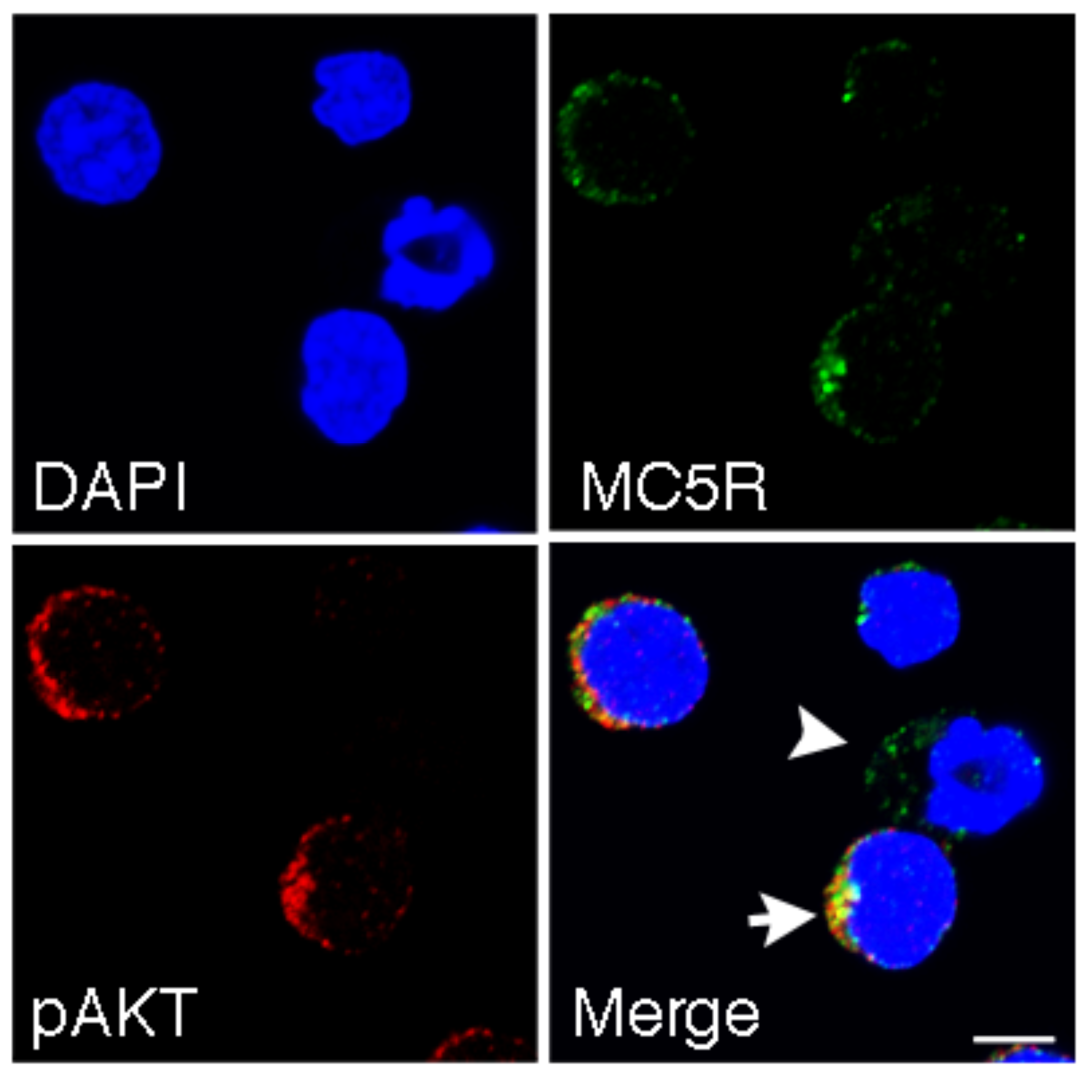

Supplement: S5 Fig — Localisation of p-AKT overlaps with that of MC5R in the periphery of cells. Arrow, P-AKT accumulated- cells expressed MC5R; Arrow head, p-AKT negative cells did not express MC5R. Bar, 5 μm. (TIF) [file pone.0123232.s005.tif]
