# Supplementary material for: Melanocortins Contribute to Sequential Differentiation and Enucleation of Human Erythroblasts via Melanocortin Receptors 1, 2 and 5
Source: PLoS One. 2015 Apr 10;10(4):e0123232. doi: 10.1371/journal.pone.0123232 (PMC4393082; doi:10.1371/journal.pone.0123232)
Supplement: S1 Table — ACTH39 indicates the full-length of the ACTH peptide. ACTH24/39 indicates the concentration of the mixture of ACTH1-24 and ACTH1-39 fragments (See Materials and Methods). (DOCX) [file pone.0123232.s006.docx]

**S1 Table. The concentration of ACTH39 and ACTH24/39 measured with ELISA.**

| **ACTH peptide** | **HPGM (pM)** | **Albuminate (pM)** |
| --- | --- | --- |
| **ACTH39** | 0.8 | 0.8 |
| **ACTH24/39** | 249.3 | 148.5 |

ACTH39 indicates the full-length of the ACTH peptide. ACTH24/39 indicates the concentration of the mixture of ACTH1–24 and ACTH1–39 fragments (See Materials and Methods).
